# Fatal Mucormycosis and Aspergillosis in an Atypical Host: What Do We Know about Mixed Invasive Mold Infections?

**DOI:** 10.1155/2020/8812528

**Published:** 2020-08-25

**Authors:** Maria Tsikala-Vafea, Weibiao Cao, Adam J. Olszewski, John E. Donahue, Dimitrios Farmakiotis

**Affiliations:** ^1^Division of Infectious Diseases, Department of Internal Medicine, The Warren Alpert Medical School of Brown University, Providence, RI 02903, USA; ^2^Department of Pathology, The Warren Alpert Medical School of Brown University, Providence, RI 02903, USA; ^3^Division of Hematology-Oncology, Department of Internal Medicine, The Warren Alpert Medical School of Brown University, Providence, RI 02903, USA; ^4^Department of Neurology, The Warren Alpert Medical School of Brown University, Providence, RI 02903, USA

## Abstract

Mixed invasive mold infections (MIMIs) are considered rare. We present a case of fatal aspergillosis and mucormycosis in an elderly host with history of chronic lymphocytic leukemia (CLL) and potential mold exposures. Notably, he had no classic risk factors for IMI other than high-dose corticosteroids, which may be an important risk factor for (M)IMI, based on the current and previous reports. There is an urgent need for studies on the “net state of immunosuppression,” environmental exposure as risk factors for (M)IMIs, and noninvasive fungal diagnostics.

## 1. Introduction

Mixed invasive mold infections (MIMIs) are rarely diagnosed due not only to presumed low incidence but also to lack of noninvasive diagnostic methods with adequate sensitivity. Previous studies have implied that corticosteroid administration is an important, potentially underestimated risk factor for MIMI [1, 2]. Herein, we present a case of fatal MIMI in a host with no “classic” risk factors for IMI, other than high-dose corticosteroids.

## 2. Case Presentation

**Figure 1:**
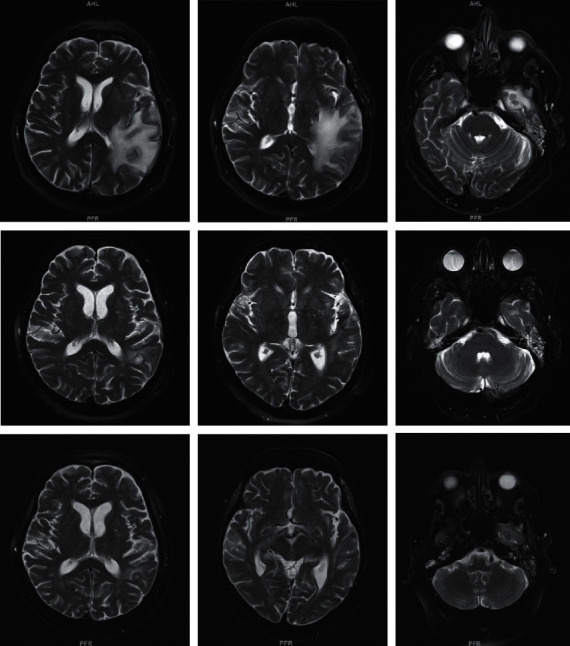
T2-weighted sequences of the brain MRI demonstrating cerebral abscesses and associated edema. Top row: initial presentation; middle row: repeat imaging, 3 weeks after initial presentation; bottom row: final imaging, 2 months after initial presentation.

**Figure 2:**
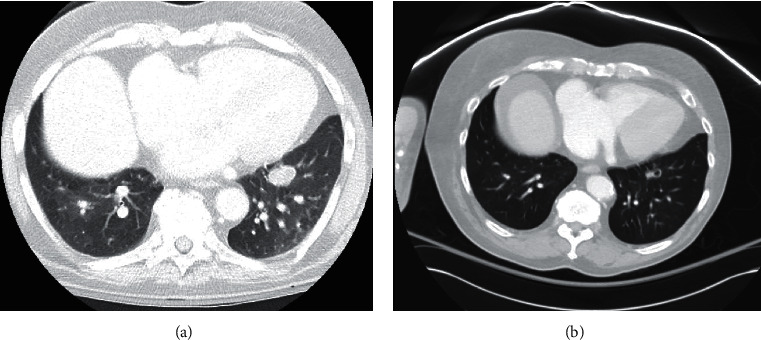
Chest CT. (a) pulmonary nodule on initial presentation; (b) pulmonary nodule on repeat imaging, 3 weeks after initial presentation.

A 79-year-old man with hypertension and chronic lymphocytic leukemia (CLL) presented with dysphasia after the first cycle of chlorambucil and obinutuzumab (anti-CD20 monoclonal antibody). He was initially diagnosed with CLL a year earlier and initiated therapy due to fatigue and progressive anemia (hemoglobin 8.9 g/dL) without prior infections. His baseline absolute neutrophil count was 2.7 × 10^9^/L, absolute lymphocyte count was 16.3 × 10^9^/L, absolute monocyte count was 0.3 × 10^9^/L, and IgG level obtained at diagnosis was 805 mg/dL. There was no history of use of corticosteroids or other immunosuppressive medications. He used to work in construction but had retired many years prior and had remained very active in his garden until initiation of chemotherapy. There was no other pertinent medical, surgical, social, or family history. Except for slurred speech, his physical exam was unremarkable. Brain MRI showed 3 distinct left temporal and parietal lobe abscesses with significant edema (Figure 1), for which he was started on high-dose dexamethasone 4 mg four times daily. Chest CT revealed a pulmonary nodule (Figure 2). Blood cultures, serum cryptococcal Ag, *Aspergillus* Ag (galactomannan, using the Platelia *Aspergillus* enzyme immunoassay (EIA)), and *β*-D-glucan (*β*Dg) were negative. Stereotactic brain biopsy of one of the abscesses failed to include its necrotic core and was nondiagnostic. Plasma cell-free DNA next-generation sequencing (cfDNA NGS) showed a strong signal for *Nocardia abscessus.* Therefore, the patient was discharged on dexamethasone 4 mg twice daily, ceftriaxone, minocycline, and linezolid, which was subsequently discontinued because of thrombocytopenia.

Repeat imaging revealed decrease in the size of the brain abscesses (Figure 1) and of the pulmonary nodule with central cavitation (Figure 2). However, he had recurrence of dysphasia and brain edema on imaging soon after tapering and discontinuation of dexamethasone approximately 6 weeks from initial presentation. He was restarted on dexamethasone 4 mg twice daily, with a plan for a slower taper.

**Figure 3:**
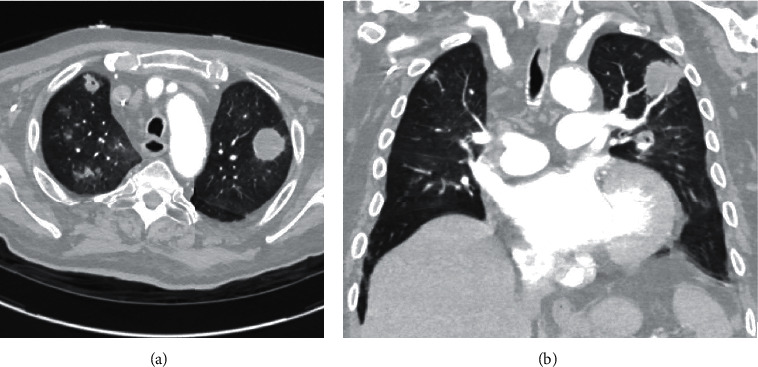
(a, b) Chest CT: invasive pulmonary aspergillosis. Chest CT findings, 2 months after initial presentation.

After two more weeks, approximately two months from his initial presentation, he was admitted to the intensive care unit with multiorgan (respiratory, renal, and heart) failure. Brain MRI showed significant decrease in the size of previous abscesses, but also many new infarcts (Figure 1). Chest CT showed multiple lung masses with cavitation, consistent with new abscesses (Figure 3). Repeat *β*Dg was negative, but galactomannan was >9.3 (EIA assay cut-off). His family opted for comfort measures only, and kindly agreed to an autopsy.

**Figure 4:**
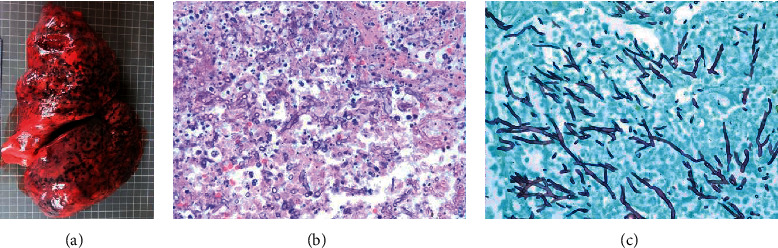
(a–c) Autopsy: invasive pulmonary aspergillosis. Left: macroscopic appearance of congested lung with infarcted and hemorrhagic areas; middle: hematoxylin-eosin (H&E) stain; right: Gomori methenamine silver (GMS) stain.

**Figure 5:**
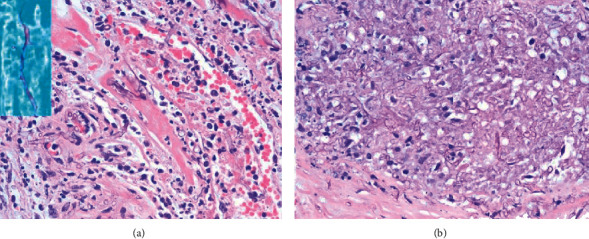
Autopsy: heart and kidneys. Cardiac (a) and renal (b) mucormycosis, H&E and GMS (small panel) stains.

**Figure 6:**
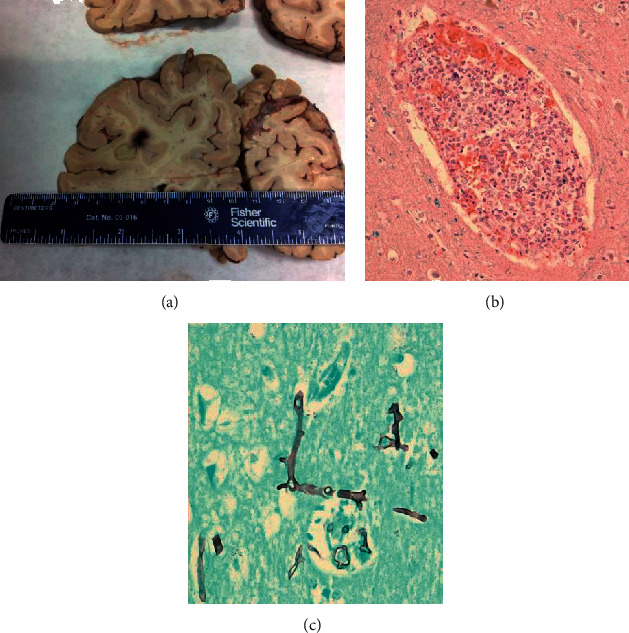
Autopsy: cerebral mucormycosis. Macroscopic appearance of temporal lobe abscess (a); abundant aseptate, wide-angled hyphae in blood vessels ((b), H&E stain) and brain parenchyma ((c), GMS stain).

**Figure 7:**
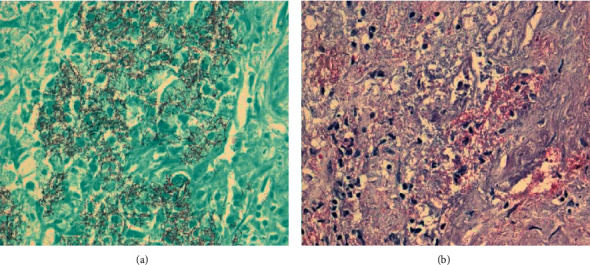
Autopsy: cerebral nocardiosis. GMS (a) and FITE (b) stains.

Autopsy showed (1) acute IMI involving both lungs. Hyphal morphology was consistent with *Aspergillus species* (septate, narrow-angled, Figure 4). Lung cultures were positive for *Aspergillus fumigatus*. (2) Acute IMI in both heart ventricles, with wide-angled, aseptate hyphae, consistent with mucormycosis (Figure 5); molecular studies (28 s/internal transcriber sequence (ITS) RNA identification, University of Washington, Seattle) came back positive for *Lichtheimia corymbifera.* (3) Cervical, mediastinal lymph node, thyroid gland, and left kidney (Figure 5) IMI. (4) Cerebral abscesses of the left temporal and parietal lobes with fungal organisms in the blood vessels and brain parenchyma, consistent with mucorales (Figure 6), and filamentous organisms identified with GMS/FITE stains, consistent with *Nocardia* (Figure 7).

## 3. Discussion

MIMIs are rarely diagnosed given lack of noninvasive diagnostic methods with adequate sensitivity; therefore, their incidence is likely underestimated. However, the associated morbidity and mortality are very high [1–9]. There are very scant data regarding MIMIs, limited to a number of case reports [2–8] and retrospective studies in patients with hematologic malignancies [1, 9]. This case report supports the hypothesis that high-dose corticosteroid administration is a risk factor for IMIs, including mucormycoses and MIMIs. Likewise, we previously reported a case of fatal mucormycosis with positive *Aspergillus* galactomannan at our institution after high-dose corticosteroids; however, that patient also had deep and likely prolonged neutropenia [2]. In the largest retrospective case series of pulmonary MIMIs to date, MIMIs were commonly diagnosed in patients with lymphoid malignancies, even in the absence of classic risk factors like neutropenia, similar to our patient. Moreover, corticosteroid administration was a significant risk factor for MIMIs [1]. Of note, in large clinical trials of patients receiving first-line CLL therapy with obinutuzumab and chlorambucil [10], there were no reported invasive fungal infections, and the overall risk of grade 3/4 infections (11%) was not significantly different from chlorambucil alone. Patients with CLL are considered immunocompromised, although opportunistic infections are typically observed in the setting of multiple lines of lymphodepleting chemotherapy and associated hypogammaglobulinemia [11].

Corticosteroids are known to increase host vulnerability to IMIs. Their immunosuppressive effects are exerted via transcriptional upregulation or repression of specific genes, mostly of the nuclear factor *κ*B (NF*κ*B) pathway. Following glucocorticoid treatment, both macrophages and neutrophils, the “front lines of defence” against invasive molds, are rendered defective. More specifically, there are impaired phagocytosis, oxidative burst-killing, impaired formation of nitric oxide, defective chemotaxis, as well as inhibition of proinflammatory cytokine production (interleukin-1, interleukin-6, TNF-a) [12].

The diagnosis of invasive fungal infections often requires invasive tissue sampling, potentially adding to the morbidity of already immunocompromised hosts. Noninvasive “fungal” markers like galactomannan and beta-D-glucan, however useful, lack ideal sensitivity for IMI and are unable to detect the mucorales. These molds are commonly drug-resistant. Therefore, such infections can progress through empiric antifungal treatment, in the absence of a specific diagnosis to help administer targeted therapy. cfDNA NGS from peripheral blood could help clinicians surpass some of these caveats. Given its ability to identify both *Aspergillus* and non-*Aspergillus* molds at the species level, it can be used in conjunction with radiographic and clinical data in order to guide optimal antifungal treatment [13–15]. However, the actual diagnostic performance of cfDNA NGS is unknown, and false negative results were not uncommon in small case-series [11, 13]. In our case, initial cfDNA NGS did not identify either of the two molds that caused fatal infection, which could speak for low overall sensitivity or, potentially, need for repeat, even serial testing.

Another understudied potential risk factor for (M)IMIs, highlighted in our case study, is environmental exposure to high mold inocula. Our patient initially did not have classic risk factors for IMIs, but used to work in construction and was very active in his garden. High-dose corticosteroids might have accelerated fungal growth and dissemination. Similarly, the case of disseminated mucormycosis that we previously reported from our institution was also linked to occupational (pipe-fitting) inhalation of spores and dissemination following high-dose corticosteroids, but also in the setting of significant neutropenia [2].

The main limitation of our case study is that we do not know if *Lichteimia* and *Aspergillus* were important culprit pathogens from early on, or secondary infections that resulted from corticosteroid administration in an elderly, “immunomodulated” host; cfDNA NGS showed a strong signal only for *Nocardia abscessus*, and nocardiosis was confirmed on autopsy. It is possible that our patient had both cerebral nocardiosis and mucormycosis. The latter may have not been detected by cfDNA NGS early on, due to the blood-brain barrier. Subsequent administration of high-dose corticosteroids may have further fueled progression of existent *Lichtheimia* infection with dissemination, along with severe pulmonary aspergillosis.

In conclusion, there is an urgent and unmet need for well-designed studies on (1) the “net state of immunosuppression” and environmental exposures as risk factors for (M)IMIs, (2) noninvasive fungal diagnostics, including serial testing by cfDNA NGS and development of new assays, and (3) the efficacy, safety, and cost-effectiveness of antimold prophylaxis in potentially susceptible hosts with characteristics other than transplant or neutropenia. Such clinical-translational research may help decrease morbidity and mortality from (M)IMIs.

## Data Availability

No data were used to support this study.
